# Clinical and laboratory differences between pediatric hospitalized patients with sickle cell disease infected or not by SARS-CoV-2

**DOI:** 10.1590/1984-0462/2023/41/2021407

**Published:** 2023-03-13

**Authors:** Gabriella Mafra Elia, Tulio Konstantyner, Rafaela Pilotto Nais, Andreia Regina Augusto dos Santos, Andrea Angel, Josefina Aparecida Pellegrini Braga

**Affiliations:** a Universidade Federal de São Paulo, Escola Paulista de Medicina, São Paulo, SP, Brazil.

**Keywords:** Sickle cell disease, Pediatrics, SARS-CoV-2, Red blood cells, Eosinophils, Doença falciforme, Pediatria, SARS-CoV-2, Hemácias, Eosinófilos

## Abstract

**Objective:**

The aim of this study was to identify clinical and complete blood count differences between pediatric hospitalized patients with sickle cell disease infected or not by SARS-CoV-2 and compare the complete blood count of patients with sickle cell disease infected by SARS-CoV-2 before hospitalization and on admission.

**Methods:**

This study was a single-center prospective cohort. Data were collected from medical records of pediatric inpatients with sickle cell disease under 18 years old infected or not with SARS-CoV-2 from the first visit to the hospital until discharge and from the last medical appointment. All patients were tested for SARS-CoV-2 by the real-time reverse transcription polymerase chain reaction.

**Results:**

Among 57 pediatric patients with sickle cell disease hospitalized from March to November 2020 in a Brazilian academic hospital, 11 (19.3%) had a positive result for SARS-CoV-2. Patients infected by SARS-CoV-2 had a higher prevalence of comorbidities than the ones who were not infected (63.6 vs. 30.4%; p=0.046). During hospital stay, no clinical or complete blood count differences between groups were found. There was a decrease in eosinophil count on hospital admission in patients with sickle cell disease infected by SARS-CoV-2 (p=0.008).

**Conclusions:**

Pediatric hospitalized patients with sickle cell disease infected by SARS-CoV-2 had more comorbidities and had a decrease in eosinophil count between hospital admission and the last medical appointment.

## RESUMO

**Objetivo:**

Identificar diferenças clínicas e laboratoriais entre pacientes pediátricos hospitalizados com doença falciforme infectados ou não por SARS-CoV-2 e comparar o hemograma completo de pacientes com doença falciforme infectados por SARS-CoV-2 antes da hospitalização e durante a admissão.

**Métodos:**

Coorte prospectiva unicêntrica, cujos dados foram coletados em prontuários de pacientes pediátricos internados com doença falciforme, menores de 18 anos, infectados ou não com SARS-CoV-2, desde a primeira visita ao hospital até a alta e desde a última consulta médica. Todos os pacientes foram testados para SARS-CoV-2 pela transcrição reversa seguida de reação em cadeia da polimerase em tempo real.

**Resultados:**

Dos 57 pacientes pediátricos com doença falciforme internados de março a novembro de 2020 em um hospital universitário brasileiro, 11 (19,3%) apresentaram resultado positivo para SARS-CoV-2. Pacientes infectados pelo SARS-CoV-2 apresentaram maior prevalência de comorbidades do que aqueles não infectados (63,6 *vs*. 30,4%; p=0,046). Durante a internação hospitalar, não foram encontradas diferenças clínicas ou laboratoriais entre os grupos. Houve diminuição da contagem de eosinófilos na admissão hospitalar em pacientes com doença falciforme infectados pelo SARS-CoV-2 (p=0,008).

**Conclusões:**

Pacientes pediátricos hospitalizados com doença falciforme infectados pelo SARS-CoV-2 apresentaram mais comorbidades e diminuição da contagem de eosinófilos entre a admissão hospitalar e a última consulta médica.

## INTRODUCTION

In December 2019, a new disease caused by SARS-CoV-2 called COVID-19 was identified in Wuhan, China.^1^ The epidemiological characteristics of COVID-19 are still being studied, but it is already known that patients with underlying conditions like immunodeficiency, chronic heart, kidney or lung disease, obesity, and other pathologies such as sickle cell disease (SCD) are among those with a higher risk of severe illness.^2,3^

SCD is a genetic disorder that results in the substitution of amino acid valine for glutamic acid in the beta-globin chain of hemoglobin. This substitution leads to changes in hemoglobin structure that will polymerize when deoxygenated and give red blood cells a sickle shape. These cells will be prematurely destroyed, leading to chronic hemolytic process and anemia, with endothelial lesion, increased adhesion molecules, hypercoagulability, and a persistent inflammatory state.^4,5^

Patients with SCD might also develop functional asplenia, which can increase the risk of infectious diseases. Viral infections, like the one caused by SARS-CoV-2, might lead to hypoxemia, acidosis, and dehydration, which can trigger vaso-occlusive crisis (VOC) and acute chest syndrome (ACS), generating a greater need for hospitalization.^6^ Patients with SCD can also have kidney disease, pulmonary hypertension, and a greater risk of thrombosis,^7^ factors that might increase the gravity of COVID-19. It is important to note that symptoms of COVID-19 such as fever, low blood oxygen levels, and dyspnea can overlap the ones in ACS, which might generate greater difficulties in recognizing and treating both diseases.

The international registry conducted by The Medical College of Wisconsin has reported 917 cases of SARS-CoV-2 infection in patients with SCD, of which the average age is 21.01 years.^8^ Data about the pediatric population is still scarce and a better understanding about how both diseases interact is needed. Therefore, our aim was to identify clinical and complete blood count (CBC) differences between pediatric hospitalized patients with SCD infected or not by SARS-CoV-2 and compare the CBC of patients with SCD infected by SARS-CoV-2 before hospitalization and on admission.

## METHOD

This study was a single-center cohort. The data were collected from medical records of inpatients. All exams were requested by the attending doctors and the follow-up period was from the first visit to the hospital until discharge. All pediatric patients under 18 years old diagnosed with SCD and hospitalized at the University Hospital in São Paulo, Brazil, from March to November 2020 were enrolled and consecutively included. All these patients were tested for SARS-CoV-2 by the reverse transcription polymerase chain reaction technique (RT-PCR). Exclusion criterion was the refusal to sign the free and informed consent term, and only one patient with SARS-CoV-2 negative was excluded.

The following data were collected: age, ethnicity, gender, weight, height, SCD genotype (SS, Sβ, Sβ^+^, and SC), previous treatment with 20–35 mg/day of hydroxyurea (HU) or chronic transfusion, and the presence of comorbidities.

Moreover, the following data were collected: respiratory (cough, rhinorrhea, or sneezing) and gastrointestinal (diarrhea, nausea, or vomiting) symptoms presented at admission and during hospital stay, CBC for groups of patients with SARS-CoV-2 positive (positive group) and SARS-CoV-2 negative (negative group) and last CBC before admission for the positive group (around 4 months), use of oseltamivir, macrolides, other antibiotics and anticoagulants, need for oxygen (oxygen saturation <93%), Intensive Care Unit (ICU), mechanical ventilation, and noninvasive ventilation. We also analyzed the following diagnoses and potential outcomes: VOC, aplastic crisis, splenic sequestration, priapism, ACS, acute renal failure, acute liver disease, venous or arterial thrombosis, stroke, septicemia, and death.

For the description of the study results, the continuous variables were expressed as median and interquartile range (IQR) and categorical variables as frequencies and 95% confidence intervals (CI).

Data consistency analysis and univariate descriptive statistics were performed for both continuous and categorical variables. In the case of comparison of continuous and categorical variables between groups with and without SARS-CoV-2, the Mann-Whitney test and the Fisher’s exact test were used, respectively. Specifically, the Wilcoxon matched-pairs signed-ranks test was used to compare laboratory test levels before and on hospital admission of children with SARS-CoV-2.

For all inferential statistics, a maximum level of p=0.05 (5%) was adopted to reject the null hypothesis. The Stata 14.2 statistics package (StataCorp, College Station, TX, USA) was used in all statistical analysis.

This study was approved by the Research Ethics Committee of the Federal University of São Paulo (Number: 34742620.6.0000.5505). All parents or guardians of the participants received information about the study and signed the consent before the study started.

## RESULTS

**Figure 1.**
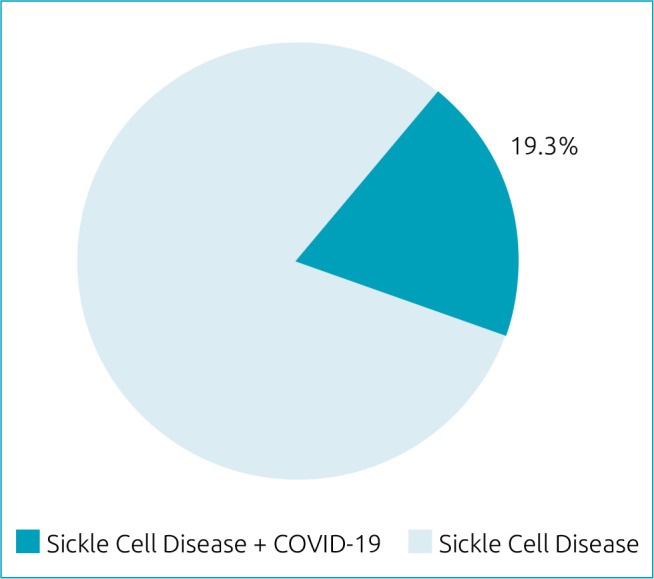
Prevalence of SARS-CoV-2 (reverse transcription polymerase chain reaction technique) in tested and hospitalized children with sickle cell disease (n=57) in São Paulo, Brazil (2020).

**Figure 2.**
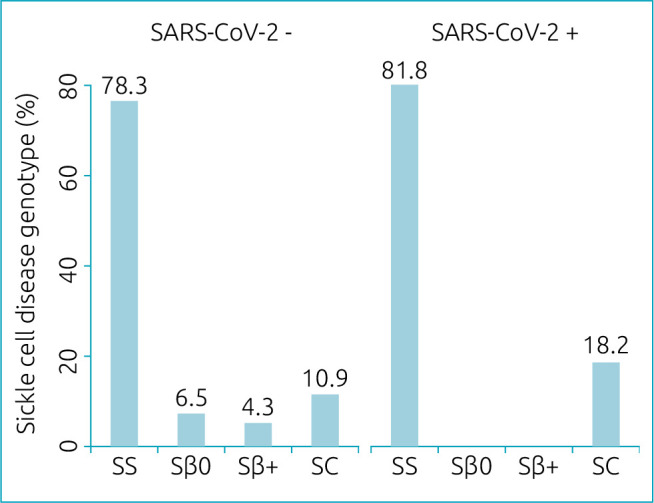
Prevalence of genotypes in tested and hospitalized children with sickle cell disease with (n=11) and without (n=46) coronavirus (SARS-CoV-2) in São Paulo, Brazil (2020).

During the recruitment period, 57 pediatric patients with SCD were hospitalized and tested for SARS-CoV-2. Among them, 11 (19.3%) had a positive result (Figure 1). The most common genotype for both the positive and negative groups was SS, and severe genotypes (SS and Sβ^0^) accounted for 81.8% in the positive group and 84.8% in the negative group (Figure 2).

Median age, ethnicity, gender, z-score of body mass index by age (BAZ), and z-score of height by age (HAZ) were similar in both groups. In the positive group, 81.8% was receiving treatment for SCD and in the negative, 63% (Table 1). During hospitalization, antibiotic and oseltamivir use was also similar (Table 1). In the SARS-CoV-2 positive group, 54.5% took HU and 27.3% were in chronic transfusion and in the negative group, 50% took HU and 13% were in chronic transfusion. The remaining patients were not receiving any of those treatments.

**Table 1.** Medians with their interquartile ranges and prevalences with their respective confidence intervals (95%CI) of clinical and therapeutic characteristics during hospitalization of children with sickle cell disease, with (+) and without (−) coronavirus (SARS-CoV-2).

Characteristics (continuous variables)	SARS-CoV-2+(n=11)		SARS-CoV-2−(n=46)		p-value*
	Median	(IQR)	Median	(IQR)	
Age (years)	10.5	(6.6–16.2)	11.0	(8.0–14.6)	0.777
Hospitalization days	6.0	(3.0–10.0)	7.0	(4.0–9.0)	0.738
BAZ	0.3	(-1.59–1.35)	-0.43	(-1.07–0.35)	0.375
HAZ	-1.03	(-2.50–-0.55)	-0.93	(-1.57–-0.39)	0.470
**Characteristics (categorical variables)**	**SARS-CoV-2 + (n=11)**		**SARS-CoV-2 − (n=46)**		**p-value^†^**
	**P (%)**	**(95%CI)**	**P (%)**	**(95%CI)**	
Non-white ethnicity	81.8	(42.0–96.5)	82.6	(68.3–91.3)	0.625
Male gender	36.4	(11.7–71.2)	41.3	(27.7–54.4)	0.522
Severe genotype	81.8	(42.0–96.5)	84.8	(70.7–92.8)	0.558
Presence of any comorbidity	63.6	(28.8–88.3)	30.4	(18.5–45.7)	0.046
Hydroxyurea or chronic transfusion	81.8	(42.0–96.5)	63.0	(47.8–76.1)	0.206
Macrolides	54.5	(22.6–83.2)	52.2	(37.4–66.6)	0.578
Other antibiotics	100	–	89.1	(75.8–95.6)	0.327
Oseltamivir	27.3	(7.2–64.6)	19.6	(10.2–34.1)	0.421

IQR: interquartile range; BAZ: z-score of body mass index by age; HAZ: z-score of height by age; P: prevalence; CI: confidence interval. The severe genotype corresponds to SS or Sβ^0^. *Mann-Whitney test; ^†^Fisher’s exact.

Underlying conditions, beside SCD, were identified in seven patients (63.6%) in the positive group, being one case of overweight, one of epilepsy, one of epilepsy and insulin resistance, one of bipolar disease, one of short stature undergoing growth hormone treatment, one of asthma and pyelocaliceal dilation, and one of asthma and left ventricular dilation. In the negative group, 14 patients (30.4%) had comorbidities. There were five cases of asthma, one case of hypertension and obesity, one of autoimmune hepatitis and microalbuminuria, one of microalbuminuria, three cases of osteoporosis or osteopenia, one of osteopenia and iron overload, one case of iron overload, and one case of left ventricular dilation and osteopenia and recurrent venous thrombosis. The prevalence of comorbidities was different between the groups (p=0.046) (Table 1).

During the hospital stay, the average days of hospitalization did not differ, being six for the positive group and seven for the negative group. Blood oxygen levels were less than 93%, and therefore the need for oxygen therapy was similar in both groups (72.7 vs. 56.5%, p=0.264). Regarding the symptoms and complications presented at admission and during hospitalization, there was no statistically significant difference among ACS, respiratory, and gastrointestinal symptoms. Specifically, fever during the hospitalization period was more frequent in the SARS-CoV-2 positive group, although without statistical significance (90.9 vs. 60.9%; p=0.055) (Table 2).

**Table 2.** Prevalences with their respective confidence intervals (95%CI) of the clinical manifestations and diagnoses on admission and during hospitalization of children with sickle cell disease, with (+) and without (−) coronavirus (SARS-CoV-2).

Clinical manifestations and diagnoses	SARS-CoV-2+(n=11)		SARS-CoV-2−(n=46)		p-value*
	P (%)	(95%CI)	P (%)	(95%CI)	
On admission					
Fever	45.5	(16.8–77.4)	28.3	(16.8–43.4)	0.226
Cough, rhinorrhea, or sneezing	18.2	(3.5–58.0)	17.4	(8.7–31.7)	0.625
Chest pain	27.3	(7.2–64.6)	19.6	(10.2–34.1)	0.421
Diarrhea, nausea, or vomiting	18.2	(3.5–58.0)	10.9	(4.4–24.2)	0.408
Vaso-occlusive crisis	63.6	(28.8–88.3)	87.0	(73.2–94.2)	0.088
During hospitalization (maintenance or recurrence)					
Fever	90.9	(46.3–99.1)	60.9	(45.7–74.2)	0.055
Cough, rhinorrhea, or sneezing	27.3	(7.2–64.6)	30.4	(18.6–45.7)	0.576
O_2_ saturation <93%	72.7	(35.4–92.8)	56.5	(41.5–70.4)	0.264
Diarrhea, nausea, or vomiting	9.1	(0.9–53.7)	6.5	(2.0–19.1)	0.587
Vaso-occlusive crisis	63.6	(28.8–88.3)	82.6	(68.3–91.3)	0.164
Acute chest syndrome	45.5	(16.8–77.4)	52.2	(37.4–66.6)	0.474

P: prevalence; CI: confidence interval. *Fisher’s exact.

In this study, as a complication of SCD, there were no cases of stroke, renal or liver impairment, sepsis, aplastic crisis, splenic sequestration, priapism, or thrombosis. One patient in the positive group was suspected of pulmonary embolism and was started on therapeutic anticoagulation. Pulmonary embolism was ruled out and anticoagulation was switched to prophylactic. No other patient was placed on the prophylactic anticoagulant during hospitalization. No patient from the positive group and one patient from the negative group was sent to the ICU due to severe ACS and need for noninvasive ventilation. No patient died or needed mechanical ventilation.

CBC was similar in both groups (Table 3). In the positive group, data from blood count and reticulocytes at admission showed no difference when compared with those at the last medical appointment, other than the eosinophil count being lower on admission (p=0.008) (Table 4).

**Table 3.** Medians with their interquartile ranges of the CBC on admission of hospitalized children with sickle cell disease, with (+) and without (−) coronavirus (SARS-CoV-2).

Laboratory tests	SARS-CoV-2+(n=11)		SARS-CoV-2−(n=46)		p-value*
	Median	(IQR)	Median	(IQR)	
Hemoglobin (g/dL)	8.8	(8.0–9.8)	8.8	(7.5–9.8)	0.686
Leukocytes (/μL)	13,518	(11,412–23,600)	16,250	(11,960–21,710)	0.628
Neutrophils (/μL)	8,787	(5,277–14,465)	9,535	(7,220–13,990)	0.856
Eosinophils (/μL)	99	(0–217)	158	(0–328)	0.330
Lymphocytes (/μL)	3,369	(1,942–5,137)	3,384	(2,613–6,320)	0.332
Monocytes (/μL)	1,745	(656–2494)	1,206	(873–1,887)	0.777
Platelets (/μL)	411,000	(230,000–449,000)	356,500	(264,000–483,000)	0.911

IQR: interquartile range. *Mann-Whitney test.

**Table 4.** Medians with their interquartile ranges of the CBC before and on admission of hospitalized children with sickle cell disease and coronavirus (SARS-CoV-2).

Laboratory tests	Before hospitalization (n=11)		On admission (n=11)		p-value*
	Median	(IQR)	Median	(IQR)	
Hemoglobin (g/dL)	9.0	(7.8–9.9)	8.8	(8.0–9.8)	0.350
Leukocytes (/μL)	11,820	(9,970–13,049)	13,518	(11,412–23,600)	0.131
Neutrophils (/μL)	6,003	(4,208–6,619)	8,787	(5,277–14,465)	0.091
Eosinophils (/μL)	366	(266–789)	99	(0–217)	0.008
Lymphocytes (/μL)	3,538	(2,661–4,925)	3,369	(1,942–5,137)	0.929
Monocytes (/μL)	1,200	(990–1,460)	1,745	(656–2494)	0.248
Platelets (/μL)	414,000	(262,000–561,000)	411,000	(230,000–449,000)	0.286
Reticulocytes (%)	8.85	(6.90–13,07)	10.72	(6.80–14.60)	0.441

IQR: interquartile range. *Wilcoxon matched-pairs signed-ranks test.

## DISCUSSION

Of the 57 patients with SCD who were admitted to the university’s hospital, 11 were confirmed to have SARS-CoV-2 infection. Baseline characteristics were balanced between patients of both groups regarding age, sex, BAZ, HAZ, genotype, ethnicity, and comorbidities. These data made it possible to compare further information about the hospitalization period since patients were homogeneous.

The higher prevalence of severe genotypes in hospitalization numbers in both positive and negative groups is compatible with the pathophysiology of SCD, since sickle cell anemia and Sβ^0^ patients have a worse clinical course during lifetime,^9,10^ with more hospitalizations, severe hemolytic anemia, and organ damage.^11^

Regular treatment with HU or chronic transfusion was also similar in both groups and did not affect the clinical outcome. However, since there was a small number of patient using those therapies in the sample, our study did not answer the question of whether these modalities have a protective effect or not. Previous literature has already established HU use in the pediatric population due to its beneficial effect on slowing down organ damage and lowering the number of hospitalizations, VOC, and ACS,^12^ but there is lack of data in literature to predict its effect in this new pandemic scenario.^13^

In adults, certain underlying medical conditions defined by the Centers for Disease Control^4^ are at increased risk of severe illness during COVID-19. Among children, evidence on which comorbidities are associated with worse clinical outcomes is still limited, but severe neurological and genetic disorders, diabetes, chronic kidney and lung diseases, immunosuppression, congenital heart disease, as well as SCD, might be associated with higher risk of ICU need, intubation, or death.^14,15^ In our study, comorbidities were more frequent in the positive group. Although there was a significant difference, it is not possible to assign to specific comorbidities a higher risk of having severe infection by SARS-CoV-2 in SCD pediatric patients due to the small sample size and the diversity of underlying medical conditions found in this study. Also, when it came to the clinical course, both groups had similar outcomes and it was not possible to predict the impact on the gravity of COVID-19. Like the SCD registry,^8^ asthma is one of the most common comorbidities in patients with COVID-19.

As for the therapy used, oxygen supply was similar in both groups. COVID-19 has already been related to low levels of oximetry and a high need for oxygen supply.^16^ Our data showed that in the positive group, the prevalence of blood oxygen level less than 93% was higher than the prevalence of ACS, a complication that can also require oxygen therapy.^17^ This might confirm SARS-CoV-2’s important role in inducing an excessive lung injury.^18^ Antibiotics were used in all COVID-19 patients; macrolides were used in almost half of these cases. Oseltamivir was used in both groups. Anticoagulation was prescribed in one case in the positive group, although this therapy is not protocol for all inpatients in our service. In adults, due to microvascular thrombosis^19^ caused by COVID-19, it is recommended to initiate prophylactic anticoagulation for inpatients. In children and adolescents, up to the present moment, there is no consensus to prescribe it in all cases, but to analyze each patient’s risk.^20^

In our study, it was not possible to identify patients who were more likely to have COVID-19 at admission since there was no statistical difference in the symptoms presented at the entrance, although the negative group presented a higher tendency to VOC. These findings are consistent with pain being a common acute manifestation of SCD and VOC, one of the most important causes of hospitalization in Brazil^21^ and worldwide.^22^ Pain has also been described as an initial presentation of COVID-19 in patients with SCD and during the clinical course of the disease.^23,24^ Secure-SCD registry^8^ showed a prevalence of pain in children aged below 18 years during COVID-19 of 40.6%. In our study, pain also continued to be an important manifestation among inpatients infected with SARS-CoV-2. During hospitalization, fever showed a tendency of higher prevalence in the positive group, which is consistent with the symptom being one of the most common in patients with COVID-19.^25^

Patients in both groups had a similar prevalence of ACS, and all had favorable clinical outcomes. ACS can be triggered by viral infection^6^ such as the one caused by the new coronavirus. Compared with literature, the Secure-SCD registry^8^ showed a prevalence of ACS during COVID-19 in children and adolescents under 18 years old of 14%; however, in our study the prevalence was higher.

In addition, no patient required intensive care support in the positive group, although previous data showed a higher prevalence of ICU need in pediatric patients with SCD (11.76%).^24^ One patient in the negative group was sent to the ICU due to severe ACS and need for noninvasive ventilation. There were no deaths in this study. When compared with adults, our data are consistent with literature, in which COVID-19 was shown to be less aggressive in children with SCD.^24^ Until the present moment, only 1 death was reported in an adolescent in the pediatric group and 18 in the adult group.^8^ Patients with SCD have a persistent inflammatory state,^4,5^ and since SARS-CoV-2 might trigger inflammatory response, it was expected that both pathologies would enhance the cascade.^26^

There was no difference in the concentrations of red and white cells and platelets in both groups. CBC of patients with SCD usually shows anemia, neutrophilia, and leukocytosis, and that was confirmed in our data. However, in the positive group, there was a significant decrease in eosinophil count between the last medical appointment and admission. Once eosinophils are involved in adaptive immune responses and innate immunity, with pro-inflammatory and destructive capabilities, this finding suggests that eosinophil count is a potential biological marker for COVID-19, as it is for other acute infections.^27,28^ Also, recent studies in adult patients with COVID-19 showed eosinopenia, although not in SCD patients.^29,30^ Regarding neutrophil and leukocyte count, the infected patients showed a tendency to higher values when comparing after and before admission, but there is still insufficient data in the literature to confirm these findings.

A key limitation of this study was the small number of patients, which could be an explanation for the few statistically significant differences found. Moreover, the data were collected in 2020, before the era of the Delta and Omicron variants. Although the impact of this specific virus on Brazilian cities was smaller than expected, these data are outdated and may be misleading.

In contrast, until the present moment, there were no studies in the literature comparing simultaneously pediatric inpatients with SCD with and without COVID-19 and comparing CBC in the SARS-CoV-2-positive group after and before hospital admission.

In conclusion, just based on a wide variety of underlying medical conditions, patients infected by SARS-CoV-2 had a higher prevalence of comorbidities than the ones who were not infected. No clinical or CBC differences between pediatric hospitalized patients with SCD infected or not by SARS-CoV-2 were found. However, there was a decrease in eosinophil count on hospital admission in patients with SCD infected by SARS-CoV-2, pointing to the presence of an infection. The patients in this study with SCD and COVID-19 had good evolution and it is important to emphasize that a comprehensive care must be provided to patients with SCD when infected by SARS-CoV-2.
